# Degradable and Photocatalytic Antibacterial Au-TiO_2_/Sodium Alginate Nanocomposite Films for Active Food Packaging

**DOI:** 10.3390/nano8110930

**Published:** 2018-11-08

**Authors:** Siying Tang, Zhe Wang, Penghui Li, Wan Li, Chengyong Li, Yi Wang, Paul K. Chu

**Affiliations:** 1 Department of Physics and Department of Materials Science and Engineering, City University of Hong Kong, Tat Chee Avenue, Kowloon, Hong Kong, China; siyintang2-c@my.cityu.edu.hk (S.T.); ph.li@siat.ac.cn (P.L.); wanli6-c@my.cityu.edu.hk (W.L.); 2 Food Science and Processing Research Center, Shenzhen University, Shenzhen 518060, China; 3 Shenzhen Institutes of Advanced Technology, Chinese Academy of Sciences, Shenzhen 518055, China; 4 Department of Applied Biology and Chemical Technology, The Hong Kong Polytechnic University, Kowloon, Hong Kong, China; yi.wy.wang@polyu.edu.hk; 5 Shenzhen Institute of Guangdong Ocean University, Shenzhen 518108, China; cyli@gdou.edu.cn

**Keywords:** alginate film, nanocomposites, antibacterial properties, food packaging

## Abstract

A degradable and antibacterial sodium alginate film containing functional Au-TiO_2_ nanocomposites for food packaging was successfully developed. The Au-TiO_2_ nanocomposites are synthesized hydrothermally and mixed with the alginate solution to form the film by a casting method. The Au-TiO_2_ nanocomposites enable the film with excellent visible light absorption and transfer ability with the light absorption rang covering UV–visible wavelength (300–800 nm) and induce the increase of the film water contact angle from 40° to 74°, which contributes to the film shape stability. Furthermore, compared to the TiO_2_ nanoparticle-incorporated film, the antibacterial ability of Au-TiO_2_/sodium alginate composite film is improved approximately by 60% and 50% against *Staphylococcus aureus (S. aureus)* and *Escherichia coli (E. coli)*, respectively, in light conditions. The antibacterial property of the film arises from the increased production of reactive oxygen species (ROS) induced by the surface plasmonic resonance of Au nanoparticles. The degradable and antibacterial properties render the composite film of great application potential in food packaging industry.

## 1. Introduction

Active food packaging offers new opportunities for food preservation and films derived from biopolymers have been widely used in food packaging due to their edible, renewable, and biodegradable characteristics [1,2,3,4,5]. Sodium alginate derived from brown algae is a green candidate to fabricate food packaging films because of advantages such as the long polymeric chain, easy accessibility, and nontoxicity [6]; it has widespread applications in the pharmaceutical, cosmetics, and biomedical fields [7,8,9,10].

To extend the functionality of active food packaging, nanoparticles such as metal nanoparticles (silver, gold, zinc), ZnO, TiO_2_ and CeO_2_ have been used to prepare composite functional films [11,12,13]. In particular, TiO_2_ nanoparticles (TiO_2_ NPs) have gained much attention due to their antibacterial activity, good photocatalytic performance, low cost, and environmental friendliness [14,15]. In 2014, the U. S. Food and Drug Administration (USFDA) approved the safety of TiO_2_ NPs use as food additives and for food contact substances after a safety assessment [16]. The antibacterial activity of TiO_2_ NPs mainly stems from the production of reactive oxygen species (ROS) such as hydroxyl radicals (OH•) and superoxide radical (O_2_^−^•) upon illumination with UV light with a wavelength of 380 nm or lower [17,18]. However, the wide bandgap of TiO_2_ NPs (3.0–3.2 eV) limits the light response in the UV range and so they cannot absorb and transfer visible light efficiently [19].

Incorporation of plasmonic nanoparticles, like gold (Au) and silver (Ag), into TiO_2_ nanostructures have been reported to enhance light absorption in the visible region due to strong surface plasmon resonance (SPR) excitation [19,20]. SPR excitation can also accelerate the interfacial charge transfer between Au and TiO_2_ NPs leading to higher photocatalytic activity compared to Au nanoparticles and pure TiO_2_ NPs [21,22,23]. Additionally, Au-incorporated TiO_2_ nanocomposites have been reported to show low toxicity, which renders approximately 90% eggs hatchability on the reproductive cycle of *Mytilus galloprovincialis* in an aquatic environment [24]. However, the Au nanoparticle-incorporated Au-TiO_2_ catalyst is not stable and can easily aggregate in liquid resulting in loss of catalytic efficiency and, therefore, it is important to immobilize Au-TiO_2_ nanocomposites in the solid film to prevent aggregation, maintain the catalytic efficiency, and produce the desirable photocatalytic antibacterial properties. 

Herein, Au-TiO_2_ nanocomposite-incorporated sodium alginate films are prepared and applied to active food packaging. The antibacterial activity of the Au-TiO_2_ nanocomposite films against *Staphylococcus aureus (S. aureus)* and *Escherichia coli (E. coli)* are evaluated and the photocatalytic activity is examined to elucidate the photocatalytic antibacterial mechanism. The optical and physical characteristics as well as degradation behavior of the Au-TiO_2_ nanocomposite-incorporated films are also determined.

## 2. Materials and Methods

### 2.1. Materials

Gold chloride trihydrate (HAuCl_4_ 3H_2_O, 99.99%), sodium borohydride (NaBH_4_, 96%), cetyltrimethylammonium bromide (CTAB, 99%), hydrochloric acid (HCl, 37%), ascorbic acid (AA, 99%), and titanium (IV) tetrafluoride (TiF_4_, 99%) were purchased from Sigma-Aldrich (Hong Kong, China). Sodium alginate (SA) and glycerol were obtained from Sinopharm Chemical Reagent Co., Ltd. (Shanghai, China). All the reagents were used without further purification. 

### 2.2. Experimental Methods

#### 2.2.1. Synthesis of Au Nanoparticle Colloidal Solution

The Au nanoparticle colloidal solution was synthesized by the seed growth method with some modifications [25]. Briefly, the gold nanoseeds were prepared by adding 600 μL of NaBH_4_ (10 mM) to a solution of HAuCl_4_ (0.5 mM, 5 mL) and CTAB (0.2 M, 5 mL) under vigorous stirring. The nanoseeds solution was kept at 37 °C for 2 h in an incubator before use. The working solution was prepared by adding HAuCl_4_ (5 mM, 1.2 mL), NaOH (1 M, 50 μL) and AA (10 mM, 500 μL) to CTAB (0.2 M, 6 mL) sequentially. After the solution became colorless, 12 μL of the nanoseeds solution were added rapidly into the working solution. It was mixed gently and stored in an incubator at 37 °C overnight. The Au nanoparticles were obtained by centrifugation at 10,000 rpm for 10 min to remove unreacted reagents and washing with deionized water twice. 

#### 2.2.2. Preparation of Au-TiO_2_ Nanocomposites and TiO_2_ Nanoparticles

The Au-TiO_2_ nanocomposites were prepared using the procedures described in the literature with some modification [26]; 90 μL of the aqueous TiF_4_ solution was dropped into 10 mL of the Au nanoparticle colloidal solution under vigorous stirring. The mixture was transferred to a Teflon-lined stainless-steel autoclave and the hydrothermal reaction was conducted at 180 °C for 12 h. The solution was cooled to room temperature and centrifuged at 7000 rpm for 10 min. The Au-TiO_2_ nanocomposites were washed with water three times to remove the remaining reagents. The final samples were dried at 40 °C in an oven and collected for further use. For comparison, pure TiO_2_ NPs were prepared by low-temperature controlled hydrolysis of TiCl_4_ in water [27,28].

#### 2.2.3. Film Preparation

The sodium alginate/Au-TiO_2_ nanocomposite (SAT) films were prepared by casting-solvent evaporation [3]. The SA powder was dissolved in distilled water at 70 °C for 30 min under magnetic stirring to form a homogenous solution (10%, *w*/*v*) and different amounts of the Au-TiO_2_ nanocomposite were added to the SA solution (0 and 2.5 wt%). Glycerol (10 wt% based on the content of dried matter) was added as the plasticizer [29] and the film-forming solutions were placed under vacuum for 60 min at room temperature to remove bubbles. Finally, the solutions were put on glass petri dishes and dried at 40 °C for 24 h. For comparison, the same amount of TiO_2_ NPs was mixed with the SA solution (2.5 wt%) to fabricate the sodium alginate/TiO_2_ NPs (ST) film. 

### 2.3. Microstructure Characterization

The UV–vis absorption spectra were acquired on the UV spectrophotometer (HALO DB-20 UV–VIS Double Beam Spectrophotometer, Dynamic Company, Livingston, UK) in the 300–800 nm range. High-resolution transmission electron microscopy (HR-TEM, JEOL-2100F, Tokyo, Japan) and field-emission scanning electron microscopy (FEI NOVA NANO SEM 450, Oregon, USA) were used to identify the size and shape of the nanocomposite and morphology of the films. The stability of the particles against agglomeration was evaluated by the zeta potential measurement (Model Zeta Sizer Nano ZS, Nano-ZS90, Malvern Instruments, Malvern, UK). Generally, 0.5 mL of the Au-TiO_2_ nanocomposite suspension (0.5 g/L) was transferred to a folded capillary cell with two caps (DTS 1070, Malvern Instruments, Malvern, UK) and the measurements were performed at room temperature in triplicates.

### 2.4. Water Contact Angle Test

A contact angle meter (OCA20, Dataphysics Co., Ltd., Filderstadt, Germany) was used to measure the surface contact angle. The sample (10 × 50 mm) was kept on a movable sample stage and leveled horizontally. 2 μL of distilled water was placed on the film using a micro-syringe and the contact angle was measured three times.

### 2.5. Photocatalytic Activity of the Sodium Alginate/Au-TiO_2_ Nanocomposite Film

The SAT film was dissolved in boiling water under stirring for 6 h to acquire the Au-TiO_2_ nanocomposite solution. The 0.5 mg/mL Au-TiO_2_ nanocomposite solution (10 mL) was dispersed in 20 mL of a solution containing methanol, ethanol, and 0.4 mM 5,5-dimethyl-1-pyrroline N-oxide (DMPO) frequently used for free radical trapping. The solution was stirred continuously in darkness for about 1 h to establish the absorption-desorption equilibrium and ESR spectra (JEOL FA100 Spectrometer, Eching b. München, Germany) were collected from the solution containing DMPO and nanoparticles (TiO_2_ NPs or Au-TiO_2_ nanocomposites) after exposure to light (λ > 380 nm) for a selected period of time. 

### 2.6. Antibacterial Evaluation 

The antibacterial activity of the SAT films (round film with diameter of 10 cm) was probed by examining their inhibitory effects on the growth of *E. coli* and *S. enterica* under light and dark conditions. After sequential ten-fold dilution with normal saline, the bacterial suspensions with 10^5^ and 10^6^ CFU/mL were obtained. The same amounts of bacteria (0.1 mL) were used for the Au-TiO_2_ nanocomposites and TiO_2_ NPs contained sodium alginate solution (10 mL). The light was generated from a 10 W LED and the exposure time was 20 min. The bacteria survival rate was calculated by the following equation: Survival rate (%) = N/N_0_ × 100, where N is the number of colonies forming units counted in the presence of Au-TiO_2_ nanocomposites or TiO_2_ NPs and N_0_ is the number of colonies forming units in presence of saline solution. 

### 2.7. Degradation Property

The degradation property of the film was studied by measuring the kinematic viscosity reduction of the film solution. The SA solutions with and without NPs (TiO_2_ NPs or Au-TiO_2_ nanocomposites) were exposed to one sun (ABET Sun 2000 simulator, class A, Milford, CT, USA) for 8 h each day for 35 days. The kinematic viscosity tests were performed on an automatic viscometer at 25 °C (DV–I Digital Viscometer, Brookfield Ltd., Middleboro, MA, USA) using 25 mL of the film solutions obtained on different days. 

### 2.8. Statistical Analysis

All the samples were analyzed in triplicate and the one-way analysis of variance (ANOVA) was applied to the data followed by Duncan to distinguish the treatments at *p* < 0.05. The statistical analyses were performed using the SPSS version 17.0 (SPSS Inc., Chicago, IL, USA).

## 3. Results and Discussion

### 3.1. Properties of Au NPs, TiO_2_ NPs, and Au-TiO_2_ Nanocomposites

**Figure 1:**
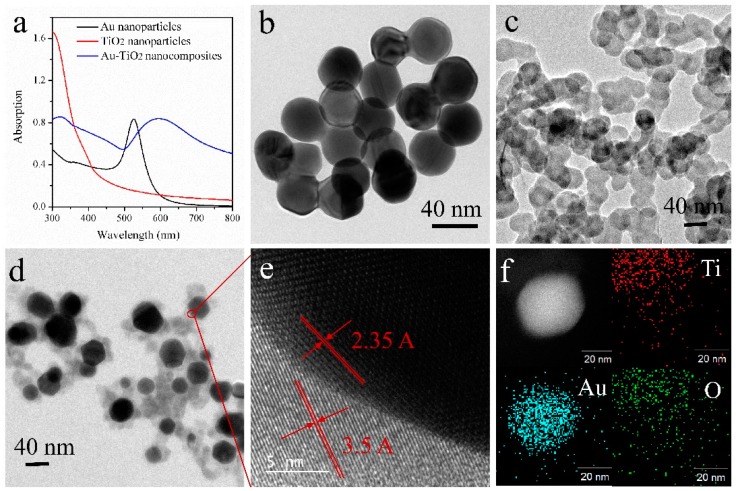
(**a**) UV–vis absorption spectra of the Au NPs, TiO_2_ NPs and Au-TiO_2_ nanocomposites films; TEM images of (**b**) Au NPs, (**c**) TiO_2_ NPs, and (**d**–**f**) Au-TiO_2_ nanocomposites.

Figure 1a shows the UV–vis absorption spectra of the Au NPs, TiO_2_ NPs and Au-TiO_2_ nanocomposites films. Light absorption by TiO_2_ NPs occurs mainly in the UV region below 380 nm, whereas that by the Au NPs is in the visible range (400–600 nm) with a peak at 512 nm. In comparison, the Au-TiO_2_ nanocomposites exhibit broad absorption from 300 to 800 nm, possibly because the valence band electrons in the TiO_2_ shell are excited at wavelengths longer than 380 nm with the aid of the Au core triggered localized energy level in the bandgap of TiO_2_ [30]. Owing to plasmonic absorption by the Au core in the Au-TiO_2_ nanocomposites, an additional absorption band (500–800 nm) is observed in the visible region. The increased visible light absorption is affected mostly by incorporation of the Au core, thereby indicating the positive effect on the visible light photocatalytic activity [31]. 

The morphology of the nanoparticles is observed by TEM. The Au NPs possess a spherical shape with a diameter of about 45 nm (Figure 1b), whereas the pure TiO_2_ NPs have a popcorn shape but poor dispersing properties (Figure 1c). The aggregation tendency of TiO_2_ NPs stems from the porous structure and surface charge. During synthesis, a large number of protons are released producing positive charges on the TiO_2_ NPs (Figure S1) [28]. Moreover, TiO_2_ NPs have a large surface area and high surface energy, and since no capping agents are attached to the TiO_2_ NPs surface, they are likely to aggregate and disperse poorly. However, during the synthesis of the Au NPs, positively charged CTAB molecules are the capping agents for the Au NPs forming a bilayer template close to the surface. CTAB is a surfactant soluble in both water and oil and the extra charges on the CTAB-coated Au NPs produce a repulsive force among the Au nanoparticles and improves the dispersity in water. Figure 1d shows the core-shell structure of the Au-TiO_2_ nanocomposites with TiO_2_ NPs surrounding the Au NPs core forming the TiO_2_ shell. Figure 1e reveals the interplanar spacings of the materials with Au (1,1,1) being 2.35 Å and anatase TiO_2_ (1,0,1) being 3.5 Å and Figure 1f confirms the elemental components in the Au-TiO_2_ nanocomposites [32].

### 3.2. Film Preparation and Characterization

**Figure 2:**
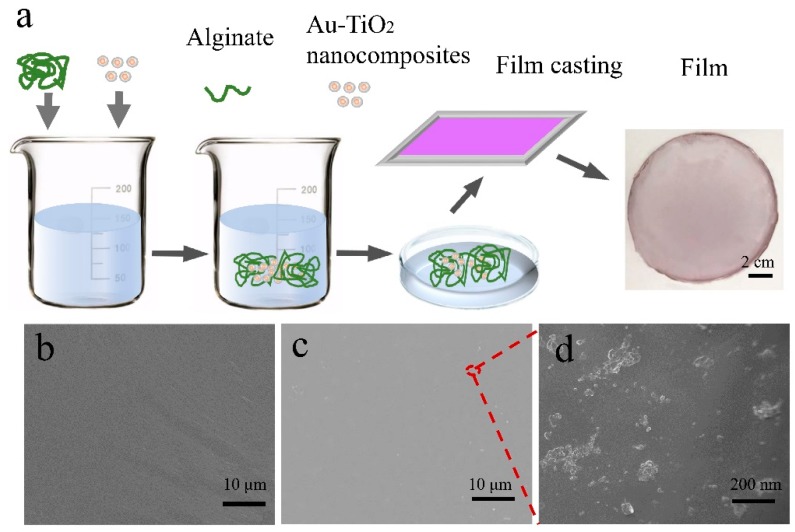
(**a)** Schematic illustration of the fabricating process of the Au-TiO_2_ nanocomposite film; SEM images of (**b**) pure alginate film and (**c**,**d**) Au-TiO_2_ nanocomposite film.

Figure 2a illustrates the fabrication process of the degradable SA/Au-TiO_2_ nanocomposite (SAT) film. The SAT film has a purple semi-transparent appearance arising from the Au-TiO_2_ nanocomposite but the SA films are transparent. For SAT films designed for packaging, transparency is an important factor. Light can go through transparent films to increase heat and photo-induced ROS affecting the contents. In comparison, the colored SAT film can block light to prevent or slow putridness and photodegradation of photosensitive nutrient contents, such as vitamin C, vitamin D and some proteins. As shown in the SEM images in Figure 2b,c, both the SA and SAT films have a smooth and continuous surface and the SAT film contains evenly distributed Au-TiO_2_ nanocomposites. The Au-TiO_2_ nanocomposites are randomly distributed on the film or imbedded in the film without destroying the film continuity (Figure 2d).

**Figure 3:**
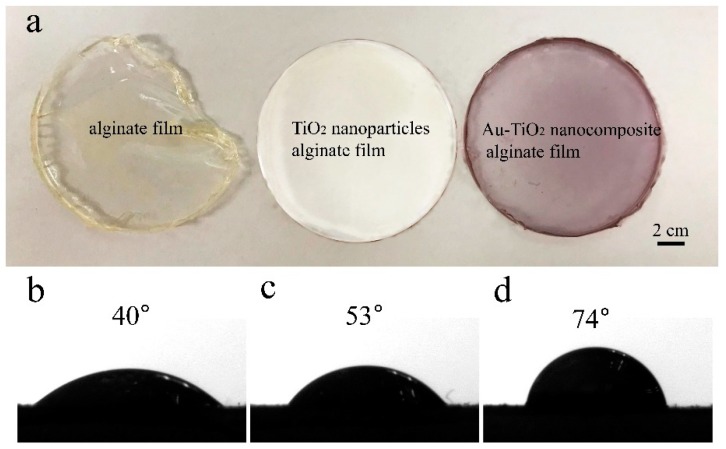
(**a**) Photos of different films after drying process; Contact angles: (**b**) Alginate film, (**c**) TiO_2_ NPs alginate film, and (**d**) Au-TiO_2_ nanocomposite alginate film.

Water sensitivity plays an important role in food packaging and the water contact angle reflects the water sensitivity. The SA films are kept at room temperature in a moist environment for one day and the photos in Figure 3a are taken after drying. Both the ST film and SAT film maintain a good shape and morphology, but the shape of the pure alginate film changes due to the reaction with water in air. The contact angle on the pure alginate film is 40° (Figure 3b) implying that the film has good affinity to water and the shape can change easily due to moisture absorption. The poor water resistance of the pure alginate film is due to the hydrophilic nature of alginate and the small amount of glycerol (plasticizer) added to enhance the film flexibility. When a certain amount of TiO_2_ NPs is added, the contact angle on the film increases to 53° (Figure 3c) and less water adsorbs onto the film. By adding the same amount of Au-TiO_2_ nanocomposites, the contact angle of the film increases further to 74° (Figure 3d). The film is less likely to be affected by shape changes caused by moisture, thus, boding well for food packaging. The improved water resistance also arises from the morphological change after addition of nanoparticles [33,34]. The results indicate that adding Au-TiO_2_ nanocomposites prevents the spreading of water drops on the film and increases the surface hydrophobicity.

### 3.3. Antibacterial Activity and Photocatalytic Antibacterial Mechanism

**Figure 4:**
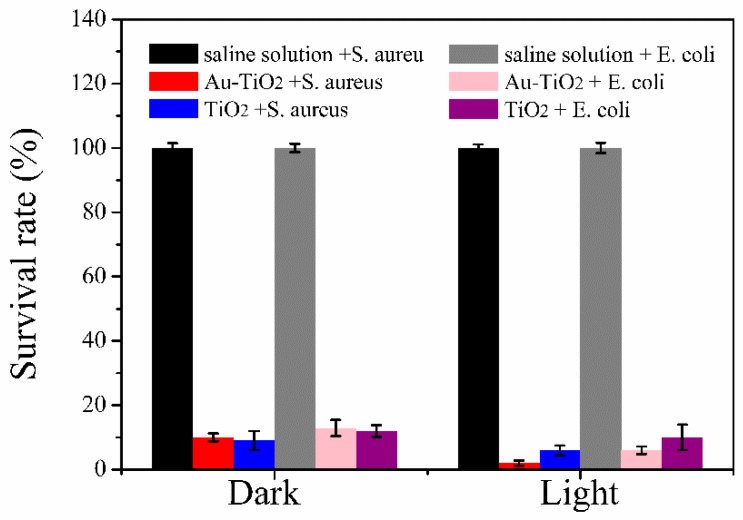
Antibacterial properties against S. aureus and E. coli under dark and visible light conditions.

As shown in Figure 4, both the SAT and ST films have good antibacterial properties against *S. aureus* and *E. coli* in the absence of light. With regard to the SAT film, the survival rates of *S. aureus* and *E. coli* are 10% and 12.9%, respectively and small differences are observed from the ST film. However, in the presence of light, the antibacterial properties of SAT against *S. aureus* and *E. coli* improve significantly but those of the ST film are enhances only slightly. The SAT film shows significantly higher (*p* < 0.05) antimicrobial efficacy than the SA film, suggesting that the Au-TiO_2_ nanocomposites have enhanced light absorption and transfer ability due to the plasmonic effect. Compared to TiO_2_ nanoparticles, incorporated nanocellulose films in the literature, the developed film presents much higher antibacterial effectiveness [35], which may be due to the SPR effect of the Au core in Au-TiO_2_ nanocomposite.

**Figure 5:**
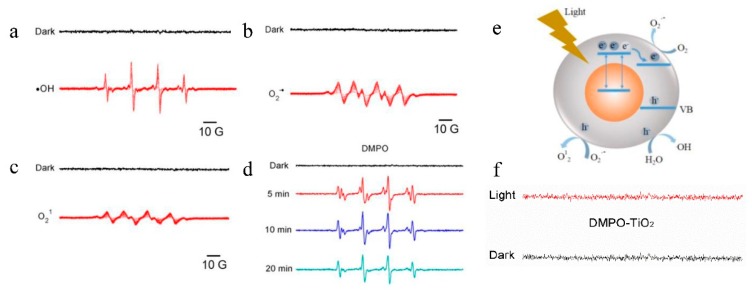
The electron spin resonance (ERS) results of (**a**) OH• radical and O_2_• radial under (**b**) dark and (**c**) light conditions; (**d**) Intensity change of OH• radical for different irradiation time; (**e**) Mechanism of radical generation; (**f**) Comparison of ERS results of the TiO_2_ nanoparticles.

The photocatalytic activity of the SAT film and resulting ROS production are believed to be the main mechanisms for the enhanced antibacterial characteristics under light irradiation. Light can activate the Au-TiO_2_ nanocomposites to produce ROS when the film is used as a packaging material with light from outside the package. Here, the photocatalytic activity is studied and ERS spectra are recorded from samples containing Au-TiO_2_ nanocomposites and spin trap (DMPO) with and without visible light (>380 nm) irradiation are shown in Figure 5. No ESR signal is observed from DMPO in the dark environment but after irradiation with visible light for 10 min, a four-line spectrum with relative intensities of 1:2:2:1 from OH• in the aqueous dispersion of DMPO is observed (Figure 5a) [33]. As shown in Figure 5d, a longer exposure time to visible light leads to increased intensity, which can be directly correlated with the abundance of DMPO-OH. Meanwhile, the maximum intensity is observed after an exposure time of 10 min and the spectrum acquired after exposure for 20 min is less intense that that after 10 min. Therefore, 10 min is the optimal exposure time and adopted in the subsequent study. O_2_^−^•/HO_2_• radicals are detected from methanol and ethanol and have smaller intensities in the characteristic signal of 1:1:1:1 (Figure 5b,c). Similarly, there is no signal under dark conditions, showing that visible light triggers the generation of radicals on the Au-TiO_2_ nanocomposites. For comparison, no ROS signals are observed under both dark and light conditions from the TiO_2_ NPs, indicating that the Au NPs help to harvest and transfer the photons to the Au-TiO_2_ nanocomposites.

Figure 5e shows that the formation of oxygen radicals under light irradiation originates from electron transfer of the semiconductor. When a semiconductor is irradiated with light with energy no less than the bandgap, electrons on the valence band can be excited to the conduction band to create charge carries, namely holes and electrons. The holes and electrons possess strong oxidative and reductive ability, respectively, leading to easy formation of ROS in aqueous media. However, TiO_2_ nanoparticles have a bandgap of 3.2 eV at a pH of 7 and so absorb UV light. Owing to the contact with the Au nanostructure, separation of electrons and holes and generation of ROS under visible light irradiation become easier and more efficient. Firstly, Au has a lower Fermi level compared to E_C_ (conductive energy level) of TiO_2_ and therefore, it can contribute to the capture of electrons from the TiO_2_ conduction band and enhance separation of electrons and holes to produce ROS. Secondly, visible light can produce the SPR effect on the Au nanoparticles resulting in hot electrons or local electrical field enhancement. Accordingly, both hot electrons and local electrical field enhancement enhance production of ROS [25]. 

Based on our results, the main factors responsible for the antibacterial properties of the Au-TiO_2_-incorporated composite films are as follows. First of all, incorporation of Au-TiO_2_ nanocomposites enhances the surface hydrophobicity of the films. Secondly, the nanostructure increases the contact area with microorganisms. Thirdly, photoexcitation of electrons in TiO_2_ NPs scarcely occurs, but the Au core is aided by visible light absorption in the Au-TiO_2_ nanocomposite and the SPR effect contributes to electron transfer from the valence state to conduction state of TiO_2_. The ROS produced enhance the antibacterial efficiency of the SAT films compared to the ST films.

### 3.4. Degradation Properties of Au-TiO_2_/Sodium Alginate Composites

**Figure 6:**
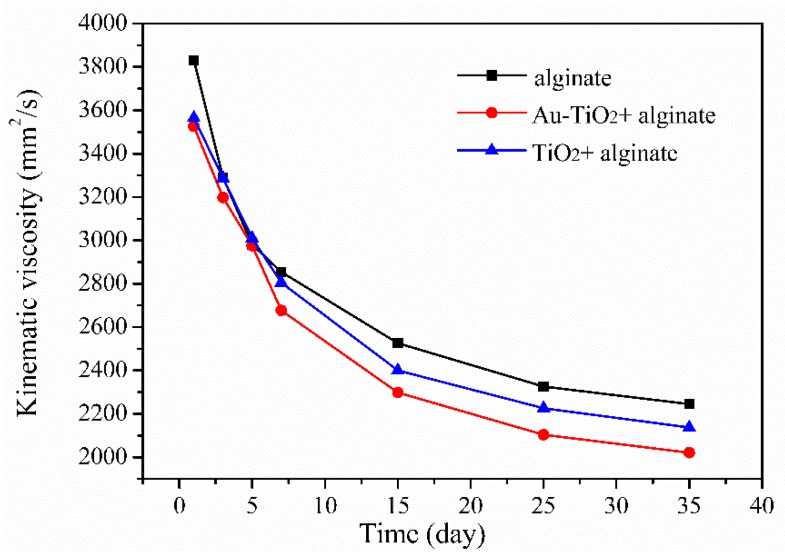
Degradation curve of the pure alginate and Au-TiO_2_ nanocomposites added alginate solutions.

According to the Mark–Houwink equation, the average molecules in sodium alginate exhibit a positive correlation with the solution viscosity at a certain concentration [36]. Figure 6 shows that the pure alginate solution degrades very fast in the beginning, but degradation slows 15 days later. In comparison, the alginate solution with Au-TiO_2_ nanocomposites degrades slowly initially due to poor water adsorption. Moreover, the viscosity of the alginate solution with the Au-TiO_2_ nanocomposite is lower than that of the pure alginate solution after exposure to sunlight for 35 days, indicating that residuals in the alginate solution with the Au-TiO_2_ nanocomposite have shorter chains and smaller molecular weight than those in the pure alginate solution. Therefore, the degradation effect of the alginate solution is improved by adding the Au-TiO_2_ nanocomposite.

## 4. Conclusions

A degradable alginate film containing Au-TiO_2_ nanocomposites is prepared. It has the ability to adsorb and transfer visible light in addition to the shape stability, antibacterial properties, and easy degradation under sunlight. The Au-TiO_2_ nanocomposites were synthesized hydrothermally and mixed with the alginate solution to form films by the casting method. The excellent properties can be attributed to surface plasmonic resonance of Au nanoparticles enhancing light absorption and conversion. Visible light produces free radicals on the Au-TiO_2_ nanocomposites giving rise to improved antibacterial properties under light irradiation, and degradation is enhanced. 

## Supplementary Materials

The following are available online at http://www.mdpi.com/2079-4991/8/11/930/s1, Figure S1: Zeta potentials of Au nanoparticles, TiO_2_ nanoparticles, and Au-TiO_2_ nanocomposites.

## References

[1] de Moraes Crizel T., de Oliveira Rios A., Alves V.D., Bandarra N., Moldão-Martins M., Flôres S.H. (2018). Active food packaging prepared with chitosan and olive pomace. Food Hydrocolloid..

[2] Arfat Y.A., Ejaz M., Jacob H., Ahmed J. (2017). Deciphering the potential of guar gum/Ag-Cu nanocomposite films as an active food packaging material. Carbohydr. Polym..

[3] Wang Z., Narciso J., Biotteau A., Plotto A., Baldwin E., Bai J. (2014). Improving storability of fresh strawberries with controlled release chlorine dioxide in perforated clamshell packaging. Food Bioprocess Technol..

[4] Giosafatto C., Pierro P., Gunning A., Mackie A., Porter R., Mariniello L. (2014). Trehalose-containing hydrocolloid edible films prepared in the presence of transglutaminase. Biopolymers.

[5] Feng Z., Wu G., Liu C., Li D., Jiang B., Zhang X. (2018). Edible coating based on whey protein isolate nanofibrils for antioxidation and inhibition of product browning. Food Hydrocolloid..

[6] Shankar S., Wang L.-F., Rhim J.-W. (2016). Preparations and characterization of alginate/silver composite films: Effect of types of silver particles. Carbohydr. Polym..

[7] Holkem A.T., Raddatz G.C., Nunes G.L., Cichoski A.J., Jacob-Lopes E., Grosso C.R.F., de Menezes C.R. (2016). Development and characterization of alginate microcapsules containing Bifidobacterium BB-12 produced by emulsification/internal gelation followed by freeze drying. LWT Food Sci. Technol..

[8] Li J., He J., Huang Y., Li D., Chen X. (2015). Improving surface and mechanical properties of alginate films by using ethanol as a co-solvent during external gelation. Carbohydr. Polym..

[9] Sarkar S., Chakraborty S., Bhattacharjee C. (2015). Photocatalytic degradation of pharmaceutical wastes by alginate supported TiO_2_ nanoparticles in packed bed photo reactor (PBPR). Ecotoxicol. Environ. Saf..

[10] Wang H.-D., Chen C., Huynh P., Chang J.-S. (2015). Exploring the potential of using algae in cosmetics. Bioresour. Technol..

[11] Chen H., Seiber J.N., Hotze M. (2014). ACS select on nanotechnology in food and agriculture: A perspective on implications and applications. J. Agric. Food Chem..

[12] Fasciani C., Silvero M.J., Anghel M.A., Arguello G.A., Becerra M.C., Scaiano J.C. (2014). Aspartame-stabilized gold–silver bimetallic biocompatible nanostructures with plasmonic photothermal properties, antibacterial activity, and long-term stability. J. Am. Chem. Soc..

[13] Sturaro A., Rella R., Parvoli G., Ferrara D., Tisato F. (2006). Contamination of dry foods with trimethyldiphenylmethanes by migration from recycled paper and board packaging. Food Addit. Contam..

[14] Goudarzi V., Shahabi-Ghahfarrokhi I. (2018). Photo-producible and photo-degradable starch/TiO_2_ bionanocomposite as a food packaging material: Development and characterization. Int. J. Biol. Macromol..

[15] Youssef A.M., El-Sayed S.M., Salama H.H., El-Sayed H.S., Dufresne A. (2015). Evaluation of bionanocomposites as packaging material on properties of soft white cheese during storage period. Carbohydr. Polym..

[16] United States Food and Drug Administration (2014). Guidance for Industry: Assessing the Effects of Significant Manufacturing Process Changes, Including Emerging Technologies on the Safety and Regulatory Status of Food Ingredients and Food Contact Substances, Including Food Ingredients That Are Color Additives. https://www.fda.gov/Food/GuidanceRegulation/GuidanceDocumentsRegulatoryInformation/IngredientsAdditivesGRASPackaging/ucm300661.htm.

[17] Deepagan V., You D.G., Um W., Ko H., Kwon S., Choi K.Y., Yi G.-R., Lee J.Y., Lee D.S., Kim K. (2016). Long-circulating Au-TiO_2_ nanocomposite as a sonosensitizer for ROS-mediated eradication of cancer. Nano Lett..

[18] Fu L., Hamzeh M., Dodard S., Zhao Y.H., Sunahara G.I. (2015). Effects of TiO_2_ nanoparticles on ROS production and growth inhibition using freshwater green algae pre-exposed to UV irradiation. Environ. Toxicol. Pharmacol..

[19] Zhang D., Wen M., Zhang S., Liu P., Zhu W., Li G., Li H. (2014). Au nanoparticles enhanced rutile TiO_2_ nanorod bundles with high visible-light photocatalytic performance for NO oxidation. Appl. Catal. B Environ..

[20] Clavero C. (2014). Plasmon-induced hot-electron generation at nanoparticle/metal-oxide interfaces for photovoltaic and photocatalytic devices. Nat. Photonics.

[21] Liu E., Fan J., Hu X., Hu Y., Li H., Tang C., Sun L., Wan J. (2015). A facile strategy to fabricate plasmonic Au/TiO_2_ nano-grass films with overlapping visible light-harvesting structures for H_2_ production from water. J. Mater. Sci..

[22] Naldoni A., D’Arienzo M., Altomare M., Marelli M., Scotti R., Morazzoni F., Selli E., Dal Santo V. (2013). Pt and Au/TiO_2_ photocatalysts for methanol reforming: Role of metal nanoparticles in tuning charge trapping properties and photoefficiency. Appl. Catal. B Environ..

[23] Wang Y.-G., Yoon Y., Glezakou V.-A., Li J., Rousseau R. (2013). The role of reducible oxide–metal cluster charge transfer in catalytic processes: New insights on the catalytic mechanism of CO oxidation on Au/TiO_2_ from ab initio molecular dynamics. J. Am. Chem. Soc..

[24] Scuderi V., Impellizzeri G., Romano L., Scuderi M., Bergum K., Zimbone M., Sanz R., Buccheri M., Simone F., Nicotra G. (2014). An enhanced photocatalytic response of nanometric TiO_2_ wrapping of Au nanoparticles for eco-friendly water applications. Nanoscale.

[25] Li N., Zhao P., Astruc D. (2014). Anisotropic gold nanoparticles: Synthesis, properties, applications, and toxicity. Angew. Chem..

[26] Zhang N., Liu S., Fu X., Xu Y.-J. (2011). Synthesis of M@ TiO_2_ (M= Au, Pd, Pt) core–shell nanocomposites with tunable photoreactivity. J. Phys. Chem. C.

[27] Abbasi S., Zebarjad S.M., Baghban S.N., Youssefi A. (2015). Synthesis of TiO_2_ nanoparticles and decorated multiwalled carbon nanotubes with various content of rutile titania. Synth. React. Inorg. Met. Org. Nano-Met. Chem..

[28] Azeez F., Al-Hetlani E., Arafa M., Abdelmonem Y., Nazeer A.A., Amin M.O., Madkour M. (2018). The effect of surface charge on photocatalytic degradation of methylene blue dye using chargeable titania nanoparticles. Sci. Rep..

[29] Gao C., Pollet E., Avérous L. (2017). Properties of glycerol-plasticized alginate films obtained by thermo-mechanical mixing. Food Hydrocolloid..

[30] Priebe J.B., Radnik J.R., Lennox A.J., Pohl M.-M., Karnahl M., Hollmann D., Grabow K., Bentrup U., Junge H., Beller M. (2015). Solar hydrogen production by plasmonic Au–TiO_2_ catalysts: Impact of synthesis protocol and TiO_2_ phase on charge transfer efficiency and H2 evolution rates. ACS Catal..

[31] Wang P., Huang B., Dai Y., Whangbo M.-H. (2012). Plasmonic photocatalysts: Harvesting visible light with noble metal nanoparticles. Phys. Chem. Chem. Phys..

[32] Haruta M. (2002). Catalysis of gold nanoparticles deposited on metal oxides. Cattech.

[33] Vejdan A., Ojagh S.M., Adeli A., Abdollahi M. (2016). Effect of TiO_2_ nanoparticles on the physico-mechanical and ultraviolet light barrier properties of fish gelatin/agar bilayer film. LWT-Food Sci. Technol..

[34] He W., Kim H., Wamer W., Melka D., Callahan J., Yin J. (2014). Photogenerated reactive oxygen species and charge carriers in ZnO/Au hybrid nanostructures are correlated with enhanced photocatalytic and antibacterial activity. J. Am. Chem. Soc..

[35] El-Wakil N., Hassan E., Abou-Zeid R., Dufresne A. (2015). Development of wheat gluten/nanocellulose/titanium dioxide nanocomposites for active food packaging. Carbohydr. Polym..

[36] Voo W.-P., Lee B.-B., Idris A., Islam A., Tey B.-T., Chan E.-S. (2015). Production of ultra-high concentration calcium alginate beads with prolonged dissolution profile. RSC Adv..

