# Supplementary material for: Degradable and Photocatalytic Antibacterial Au-TiO2/Sodium Alginate Nanocomposite Films for Active Food Packaging
Source: Nanomaterials (Basel). 2018 Nov 8;8(11):930. doi: 10.3390/nano8110930 (PMC6266112; doi:10.3390/nano8110930)
Supplement: Supplementary file 1 [file nanomaterials-08-00930-s001.pdf]

Article

Degradable and Photocatalytic Antibacterial Au-TiO_2/Sodium Alginate Nanocomposite Films for Active Food Packaging

Siying Tang [1], Zhe Wang [2,3,4,*], Penghui Li [1,3], Wan Li [1], Chengyong Li [5], Yi Wang [4] and Paul K. Chu [1,*]

[1] Department of Physics and Department of Materials Science and Engineering, City University of Hong Kong, Tat Chee Avenue, Kowloon, Hong Kong, China; siyintang2-c@my.cityu.edu.hk (S.T.); ph.li@siat.ac.cn (P.L.); wanli6-c@my.cityu.edu.hk (W.L.)
[2] Food Science and Processing Research Center, Shenzhen University, Shenzhen 518060, China
[3] Shenzhen Institutes of Advanced Technology, Chinese Academy of Sciences, Shenzhen 518055, China
[4] Department of Applied Biology and Chemical Technology, The Hong Kong Polytechnic University, Kowloon, Hong Kong, China; yi.wy.wang@polyu.edu.hk
[5] Shenzhen Institute of Guangdong Ocean University, Shenzhen 518108, China; cyli@gdou.edu.cn
* Correspondence: wangzhejida2008@163.com (Z.W.); paul.chu@cityu.edu.hk (P.K.C.); Tel: +852-3442-7724 (P.K.C.)

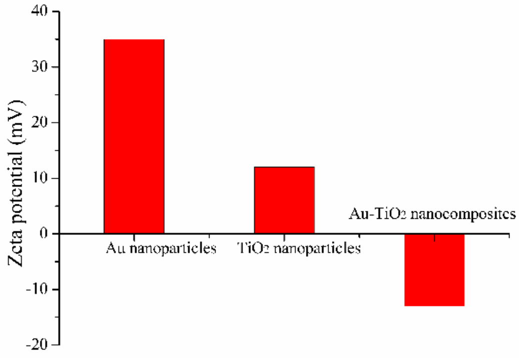

Figure S1. Zeta potentials of Au nanoparticles, TiO_2 nanoparticles, and Au-TiO_2 nanocomposites.
